# Host Modulators of H1N1 Cytopathogenicity

**DOI:** 10.1371/journal.pone.0039284

**Published:** 2012-08-02

**Authors:** Samuel E. Ward, Hyun Seok Kim, Kakajan Komurov, Saurabh Mendiratta, Pei-Ling Tsai, Mirco Schmolke, Neal Satterly, Balaji Manicassamy, Christian V. Forst, Michael G. Roth, Adolfo García-Sastre, Katarzyna M. Blazewska, Charles E. McKenna, Beatriz M. Fontoura, Michael A. White

**Affiliations:** 1 Department of Cell Biology, University of Texas Southwestern Medical School, Dallas, Texas, United States of America; 2 Divisions of Experimental Hematology and Cancer Biology, Human Genetics and Biomedical Informatics, Cincinnati Children's Hospital Medical Center, Cincinnati, Ohio, United States of America; 3 Department of Microbiology, Mount Sinai School of Medicine, New York, New York, United States of America; 4 Department of Clinical Sciences, University of Texas Southwestern Medical Center, Dallas, Texas, United States of America; 5 Department of Biochemistry, University of Texas Southwestern Medical Center, Dallas, Texas, United States of America; 6 Department of Medicine, Division of Infectious Diseases, Mount Sinai School of Medicine, New York, New York, United States of America; 7 Global Health and Emerging Pathogens Institute, Mount Sinai School of Medicine, New York, New York, United States of America; 8 Institute of Organic Chemistry, Technical University of Łódź, Łódź, Poland; 9 Department of Chemistry, University of Southern California, Los Angeles, California, United States of America; Johns Hopkins University – Bloomberg School of Public Health, United States of America

## Abstract

Influenza A virus infects 5–20% of the population annually, resulting in ∼35,000 deaths and significant morbidity. Current treatments include vaccines and drugs that target viral proteins. However, both of these approaches have limitations, as vaccines require yearly development and the rapid evolution of viral proteins gives rise to drug resistance. In consequence additional intervention strategies, that target host factors required for the viral life cycle, are under investigation. Here we employed arrayed whole-genome siRNA screening strategies to identify cell-autonomous molecular components that are subverted to support H1N1 influenza A virus infection of human bronchial epithelial cells. Integration across relevant public data sets exposed druggable gene products required for epithelial cell infection or required for viral proteins to deflect host cell suicide checkpoint activation. Pharmacological inhibition of representative targets, RGGT and CHEK1, resulted in significant protection against infection of human epithelial cells by the A/WS/33 virus. In addition, chemical inhibition of RGGT partially protected against H5N1 and the 2009 H1N1 pandemic strain. The observations reported here thus contribute to an expanding body of studies directed at decoding vulnerabilities in the command and control networks specified by influenza virulence factors.

## Introduction

The Orthomyxoviridae family member influenza A virus is the causal agent of acute respiratory tract infections suffered annually by 5–20% of the human population. There is a significant impact on morbidity, concentrated in people younger than 20 years, with economic consequences running into the billions of dollars during large epidemics [1]. In addition, viral infections are associated with development of chronic asthma and disease exacerbation in both children and adults. In particular, acute influenza infection can amplify airway inflammation in asthmatic patients and induce alterations in epithelial and stromal cell physiology contributing to allergen sensitization, exaggerated bronchoconstriction, and remodeling of airway epithelia [2]. Mortality rates associated with seasonal flu are low, but the aging population is at risk for development of severe congestive pneumonia which kills ∼35,000 people each year in the U.S. [1]. Of continual concern is the threat of emergent high virulence strains such as the Spanish flu (H1N1), Asian flu (H2N2) and Hong Kong flu (H3N2) pandemics which claimed millions of lives world-wide.

Current treatments are focused on vaccines and drugs that target viral proteins. However, both of these approaches have limitations as vaccines require yearly development and lag detection of new strains, while viral proteins have a stunning capacity to evolve resistance to targeted agents [3]. The genome of the influenza A virus consists of 8 negative single-strand RNA segments that encode 11 functional peptides necessary for viral replication and virulence [1]. Thus the viral-autonomous repertoire of gene products is extremely limited and influenza A replication is dependent upon hijacking host-cell biological systems to facilitate viral entry, replication, assembly, and budding. The recognition that a suit of human host proteins are required for IVA infection and replication presents additional targeting strategies that may be less prone to deflection by the highly plastic viral genome.

Here we have employed the cytopathic effects of H1N1 infection in bronchial epithelial cells as a mechanism to isolate host genes that represent intervention target opportunities by virtue of their contribution to H1N1 infection and replication, or by virtue of their contribution to viral virulence factor-dependent evasion of innate immune responses. A primary whole-genome arrayed siRNA screen identified gene depletions that either deflected or promoted bronchial epithelial cell death upon exposure to the H1N1 A/WSN/33 influenza virus and were not cytotoxic to mock infected cells. Integration with orthogonal data sets, describing host gene function [4]–[8], parsed collective ‘targets’ into four functional classes. 1) Targets that, when depleted, enhance bronchial epithelial cell survival upon H1N1 exposure, and are required for viral replication. This class presumably represents host factors that facilitate viral infection and/or are required to support viral replication. 2) Targets that, when depleted, reduce bronchial epithelial cell survival upon H1N1 exposure, and are required for viral replication. This important and initially unanticipated class, likely represents proviral host factors that deflect cell death checkpoint responses that would otherwise engage upon detection of viral infection. 3) Targets that, when depleted, reduce bronchial epithelial cell survival upon H1N1 exposure and enhance viral replication relative to controls. Recently discovered innate immune pathway components, such as IFITM3 that are responsive to H1N1 infection, are members of this class, which presumably represent antiviral restriction factors that normally oppose infection. 4) Targets, that when depleted, enhance bronchial epithelial cell survival upon H1N1 exposure and enhance viral replication as compared to controls. These host factors are likely responsible for influenza virus-mediated cytopathic effects. Chemical inhibition of gene products from two classes, RABGGTASE and CHEK1, indicated these targets might be pharmacologically addressable for H1N1 intervention in an epithelial cell autonomous context.

## Results and Discussion

**Figure 1:**
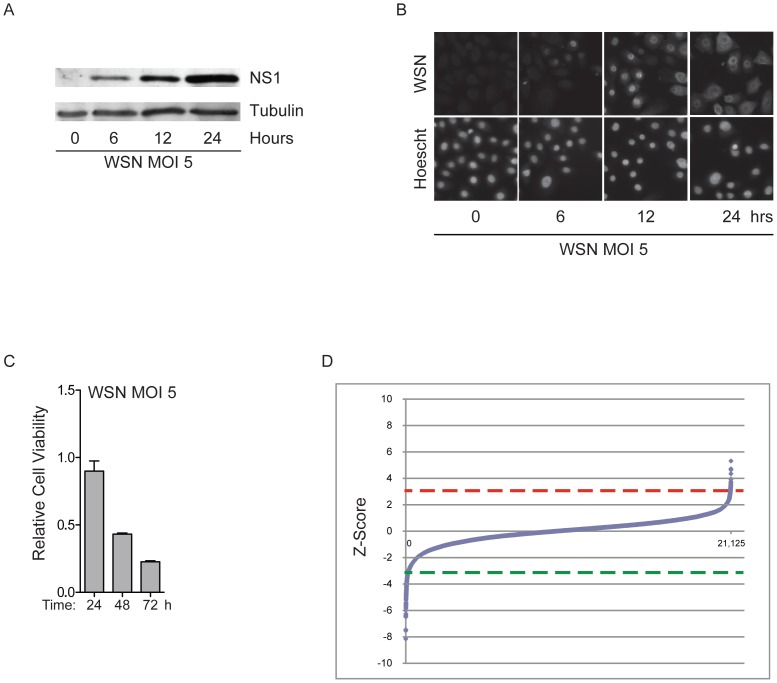
Identification of Host Modulators of Influenza Infection. (A) HBEC30 were infected with A/WSN/33/H1/N1 (WSN) at an MOI of 5 and examined for accumulation of viral proteins by immunoblot at the indicated time-points post-infection. (B) Cells treated as in B were immunostained for detection of viral protein accumulation at single cell resolution. Top panels labeled WSN show anti-influenza A staining and bottom panels labeled Hoescht show nuclear staining with Hoescht. (C) Parallel cultures were also examined for consequences on cell viability over a 72-hour time-course. (D) The rank-ordered Z-score distribution from each of 21,125 siRNA pools targeting the annotated human genome is shown. Dashed lines indicate 3 standard deviations above (red) and below (green) the mean of the distribution.

**Figure 2:**
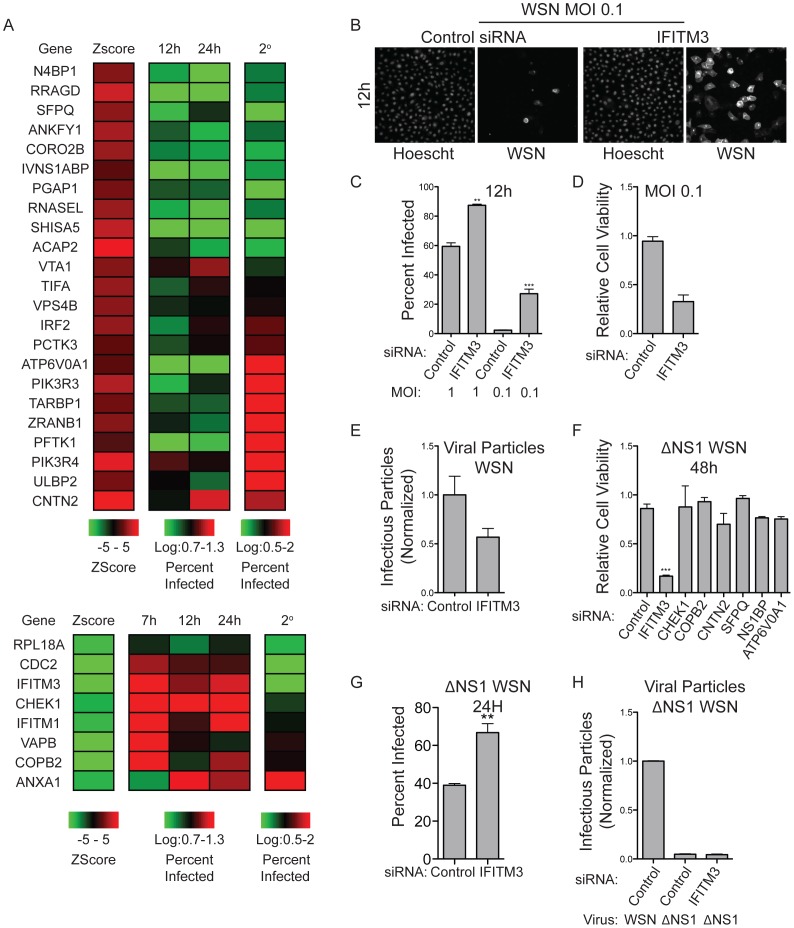
Characterization of Viral Replication Response. (A) A panel of 33 siRNAs was assayed for viral protein accumulation and infectious particle production. HBEC30s were transfected with siRNA and infected with WSN at an MOI of 5. For primary infection, cells were fixed at indicated time points and viral protein was detected by immunostaining of viral proteins. Supernatants from infected cells were collected at 24 hours post infection and used for secondary infection of MDCK cells with viral protein detection by immunostaining (right column, 2^0^). Resistors are shown in the top panel and sensitizers in the bottom. (B) HBEC30 were transfected with siRNA targeting IFITM3 or control siRNA and infected with WSN at an MOI of 0.1. Viral protein was detected at 12 hours post infection by immunostaining with anti-influenza antibodies (WSN panels) (Meridian Life Science, Inc, Cat# B65141G). (C) Cells treated in B were counted and the percent of infected cells was quantified. (D) Cells treated as in B were incubated 48 hours post infection and cell viability was measured. (E) Supernatants from WSN infected HBEC30s were collected 24 hours post infection and used for secondary infection of MDCK cells with viral protein detection by immunostaining. (F) HBEC30s were transfected with indicated siRNAs and infected with WSN lacking the viral protein NS1, cell viability was measured 48 hours post infection. (G) Cells treated as in F were fixed at 24 hours post infection and immunostained for viral protein for calculation of percentage of infected cells. (H) Supernatants from cells in G were used for secondary infection in MDCK cells and viral protein was detected by immunostaining. (P values; * <0.05, ** <0.01, *** <0.0001).

Influenza A infection is associated with pathological changes throughout the respiratory tract, however the major site of impact appears to be the respiratory epithelia. Bronchoscopy of patients with uncomplicated influenza infections reveals alterations in the ciliated epithelia of the larynx, trachea, and bronchi that includes vacuolization, loss of cilia, and desquamation of columnar epithelial cells and goblet cells down to the basal cell layer. Importantly, viral antigen is found predominantly in the epithelial cells and mononuclear cells [1]. Therefore, for the studies described here, we employed telomerase-immortalized human bronchial epithelial cells (HBEC30) that retain the capacity to differentiate into a polarized ciliated epithelial sheet [9]. In undifferentiated cell culture, we found that 100% of HBEC30 in culture display viral protein production after a 24-hour exposure to mouse-adapted virus at an MOI of 5 (Figure 1A, B). This leads to an approximately 50% decrease in cell viability 48-hours post infection (Figure 1C). Given these observations, we adopted a whole genome siRNA screening strategy that involved a 48-hour incubation post siRNA transfection, followed by a 48 hour exposure to influenza A/WSN/1933 or carrier, with cell viability as the endpoint assay. Raw viability values were converted to viability Z-scores with a metric that normalized for both position and batch effects (Figure 1D, see methods). The dynamic range of viability scores observed under screening conditions potentially affords the opportunity to identify both enhancers and resistors of viral pathogenicity (Table 1 in Supporting Information S1). Considering siRNA pools associated with Z-scores that were equal to or greater than 3 standard deviations above (resistors) or below (sensitizers) the mean of the population, 53 candidate resistors and 182 candidate sensitizers were identified (Table 2 in Supporting Information S1). A representative sample of targets was further tested for consequences on viral protein accumulation and viral replication. As might be expected, the majority of the siRNA pools that deflect a viral cytopathic response resulted in reduced viral protein accumulation, as detected by quantitation of viral proteins at single cell resolution, and reduced production of infectious particles (Figure 2A). Among these, IVNS1ABP and the splicing factor SFPQ directly interact with the viral pathogenicity factor NS1, presumably reflecting a positive role in support of viral corruption of host machinery for viral protein production [10]. Of interest in this class is RRAGD, a small G-protein that supports the amino-acid responsiveness of mTOR as a component of the “ragulator” [11]. Several reports have highlighted the importance of viral induction of mTOR for viral replication, but the mechanism is not fully elaborated [6], [12]. Given the participation of endosomes as a viral entry mechanism [13], it is tempting to speculate that RRAGD is a limiting host factor for viral corruption of mTOR regulation. Additional factors in this group are involved with the host defense response, p53-mediated cell death and vesicle maturation and trafficking. To test for false positives arising from off-target effects of siRNA treatment, we retested 88 siRNA pools as four individual oligos. Approximately 60% of siRNAs retested with two or more oligos reproducing the original phenotype (Figure S1).

Among the most potent members of the sensitizer class were the previously described proviral host factor IFITM3 and its homolog IFITM1 (Table 7 in Supporting Information S1). IFITM3 has been reported to be required for restriction of viral infection and is thought to inhibit viral entry [4], [14]. These gene products are interferon responsive, and depletion was associated with enhanced viral pathogenicity and enhanced viral protein production at limiting MOIs (1 and 0.1) as compared to controls (Figure 2A, B, C). Unexpectedly, cells depleted of IFITM3 produced fewer infection competent viral particles as determined by secondary infection of MDCK cells with cell culture supernatants (Figure 2 A, E). For these assays, HBEC30 cell cultures were infected with an MOI of 5 for 48 hours post transfection with siRNA pools. Supernatants were collected 24 hours post infection and used to infect confluent MDCK cell cultures. Notably, we observed enhanced frequency as well as enhanced amplitude of viral protein accumulation in IFITM3 depleted cells during primary infection. Reduced production of infectious particles, in the face of enhance viral protein production, may therefore be a consequence of either limiting host factors or disruption of viral protein/host factor stoichiometry required for assembly of viable viral particles. Of interest, the viral cytophathic effect was greatly enhanced upon IFITM3 depletion in the presence or absence of the virulence factor NS1, a viral protein known to block many of the innate immunity responses [15]–[19] (figure 2F, G). However, deletion of NS1 results in complete failure of infectious particle production even upon IFITM3 depletion (Figure 2H). These observations would place IFITM3 function early in the viral life cycle and independent of NS1 function, consistent with reports that indicate IFITM3's antiviral activity is at the level of viral entry [14].

**Figure 3:**
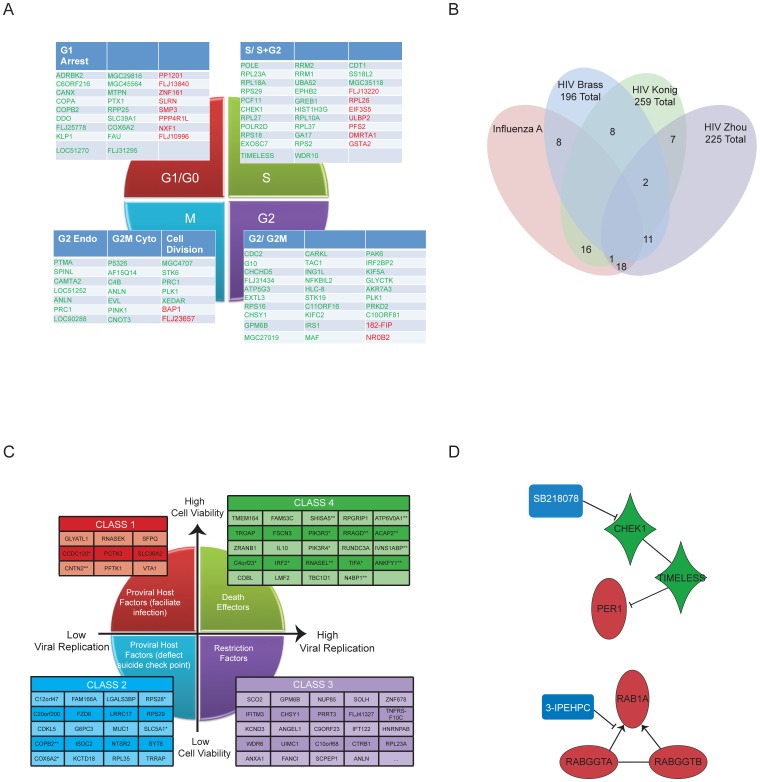
Functional Classification of Candidate Hits. (A) siRNA screen results from this study were compared with data from two published screens for cell cycle modulators and the overlap is shown. (B) Intersection of hits from this study with those examining host modulators of HIV infection (Table 4 in Supporting Information S1). (C) Cell viability data was queried against four published screens using viral replication as the end-point assay. Candidate hits were binned into functional classes based upon perturbation of viral cytopathogenicity together with viral replication. (D) Two pharmacologically addressable Netwalk subnetworks are shown.

Depletion of the cell cycle/DNA damage checkpoint proteins CDC2 and CHEK1, like IFITM3, appeared to promote viral protein production and cytopathic response, while impairing assembly of infection-competent viral particles. A global comparison of the candidate modulators of H1N1 pathogenicity identified here with two whole-genome siRNA screens for modulators of cell cycle progression revealed a significant intersection (Figure 3A). However, CDC2 and CHEK1 depletion show quite distinct consequences on G1 versus G2 arrest suggesting their contribution to H1N1 infection may be independent of cell cycle control. CHEK1 has not been previously isolated in viral pathogenicity or viral replication screens, including those performed with the same siRNA library employed here (Figure 3B, C, Tables 3 and 4 in Supporting Information S1). To investigate additional biological processes that may be associated with CHEK1 modulation of viral infection, we assembled a context-specific protein-protein interaction sub-network defined by the genomic Z-score distribution of the primary screen (Figure S7). This subnetwork revealed the circadian gene Timeless, recently defined as a master regulator of the host defense response [20], within the first-degree neighborhood of CHEK1 (Figure 3D). Given this association, we investigated the consequence of chemical inhibition of CHEK1 on H1N1 infection.

**Figure 4:**
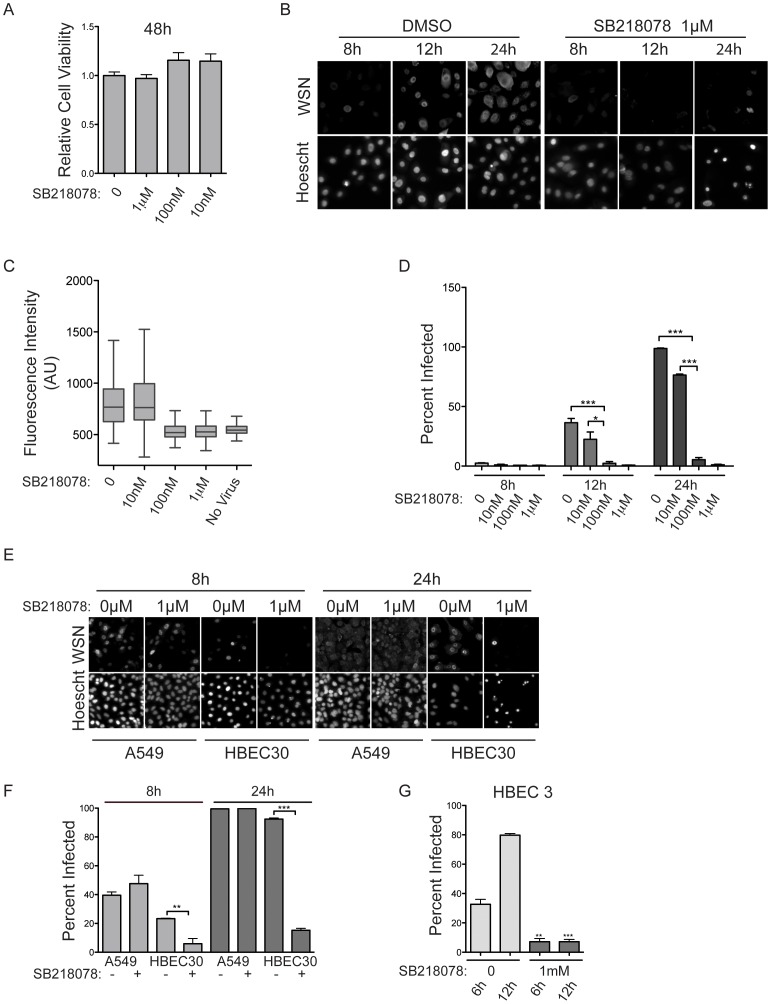
Viral Inhibition by SB218078. (A) HBEC30s were treated with SB218078 at indicated concentrations and cell viability was measured after 48 hours. (B) HBEC30s were treated as in A and infected with WSN at an MOI of 5 followed by immunostaining at indicated time points. Top panels labeled WSN show anti-influenza A staining and bottom panels labeled Hoescht show nuclear staining with Hoescht. (C) Fluorescence intensity was measured and quantified from B. (D) Percentage of infected cells from B. (E) A549 cells were pretreated with 218078 and infected with WSN at an MOI of 5. Viral protein was detected by immunostaining. Top panels show anti-influenza A staining (WSN) and bottom panels show nuclear staining (Hoescht). (F) Quantification of percent of infected cells in E. (G) HBEC3-KT cells were pretreated with SB218078, infected with WSN at an MOI of 5 and immunostained for detection of viral protein. The percentage of infected cells was quantified. (P values; * <0.05, ** <0.01, *** <0.0001).

We employed SB218078, an investigational CHEK1 inhibitor similar to one currently in clinical trials as an anti-neoplastic agent, with an in vitro IC50 of 0.015 μM and a K_i,app._ of 15±4 [21], [22]. Pretreatment of cultures with 1 uM or 100 nM SB218078 for 12 hours resulted in significant inhibition of viral protein accumulation together with a marked virus-specific death response by 24 hours (Figure 4A, B). While some viral infection was detected at the 100 nM dose, viral protein production was severely limited at single cell resolution (Figure 4B, C). These observations suggest that SB218078 is releasing a cell death response to viral detection that would otherwise be suppressed during the viral replication cycle. Viral-induced cell death was also observed upon siRNA-mediated CHEK1 depletion (Figure 2A). The seemingly contradictory increase in infection frequency upon CHEK1 depletion may therefore be an indirect consequence of infection of low density residual cell populations with hypomorphic CHEK1 activity. Remarkably, SB218078 had no consequence on H1N1 replication in A549 cells, a cancer cell line often employed to test for modulators of viral replication and host responses [5], [23], [24] (Figure 4E, F). However, a nontransformed, telomerase-immortalized bronchial epithelial cell line, derived from a different patient, HBEC3 [25], was identical to HBEC30 in its responsiveness to SB218078 (Figure 4G). These observations indicate intervention targets may be available in non-tumorigenic cells that are uncoupled from host regulatory networks in cancer cells, and potentially explain why CHEK1 was not identified in other efforts to date that have universally relied on cancer lines as screen hosts [4]–[7], [26].

We next queried the behavior of gene depletions identified here that modulate H1N1 cytopathic effects to those in 4 whole-genome siRNA screens which measured influenza virus replication as the end-point assay [4]–[7]. This allowed us to parse collective ‘hits’ into four functional classes (Figure 3C, Table 5 in Supporting Information S1). Class 1: genes that, when depleted, enhance bronchial epithelial cell survival upon H1N1 exposure, and are required for viral replication. This class presumably represents host factors that facilitate viral infection and/or are required to support viral replication. Class 2: genes that, when depleted, reduce bronchial epithelial cell survival upon H1N1 exposure, and are required for viral replication. This, initially unanticipated but very intriguing class, likely represents host factors that deflect cell death checkpoint responses that would otherwise engage upon detection of viral infection. Class 3: genes that, when depleted, reduce bronchial epithelial cell survival upon H1N1 exposure and enhance viral replication relative to controls. This class presumably represents antiviral restriction factors that normally oppose infection. Class 4: genes, that when depleted, enhance bronchial epithelial cell survival upon H1N1 exposure and enhance viral replication as compared to controls. Of note, Class 2, which may represent novel intervention target opportunities, includes TRRAP, a large multidomain protein of the phosphoinositide 3-kinase-related kinases (PIKK) family that is a component of many histone acetyltransferase (HAT) complexes. TRRAP was recently identified as a bona fide oncogene in melanoma through cancer genome resequencing efforts, however, its transforming mechanism is unknown [27].

**Figure 5:**
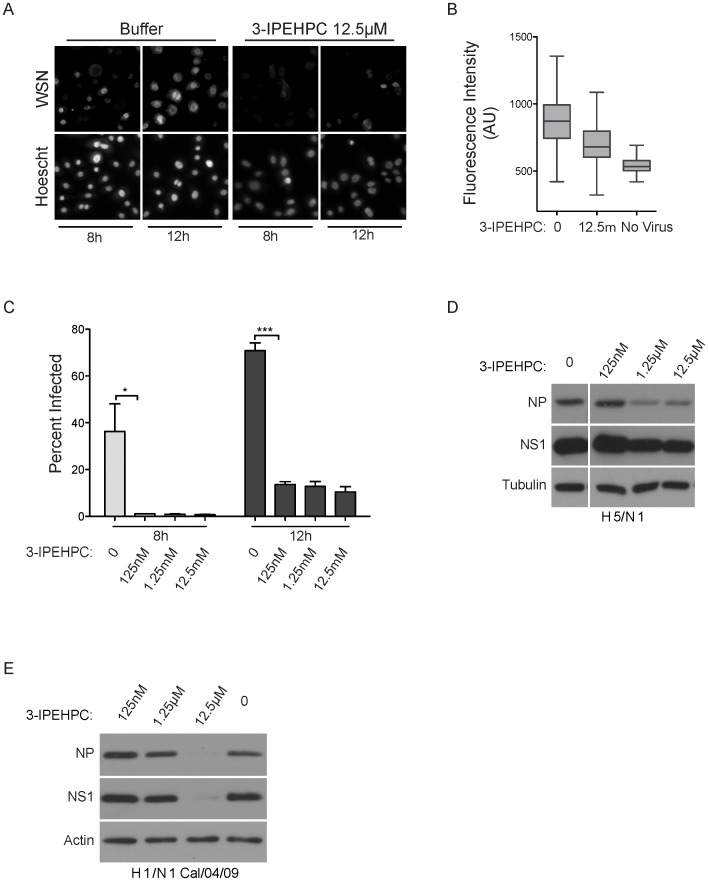
Viral Inhibition by 3-IPEHPC. (A) HBEC30s were pretreated with 3-IPEHPC or buffer and infected with WSN at an MOI of 5. Viral protein was detected by immunostaining. Top panels show anti-influenza A staining (WSN) and bottom panels show nuclear staining (Hoescht). (B) Overall fluorescence intensity of cells in A was quantified. (C) Quantification of percent of infected cells in A. (D and E) A549 cells were pretreated with 3-IPEHPC and infected with either avian H5/N1 or the recent H1/N1 pandemic strain. Lysates from infected cells were collected 24 hours post infection and viral protein was detected by immunoblot. (P values; * <0.05, ** <0.01, *** <0.0001).

By nature, a challenge to siRNA-screening efforts is false negatives that derive from weak phenotypes due to suboptimal depletion of what are otherwise key factors in the biological process under investigation. One opportunity to help meet this challenge is to employ coherent behavior of gene sets to identify key biological processes supporting a phenotype rather than relying solely on an arbitrary scoring threshold for each individual gene. We employed Netwalk [28] here to facilitate identification of such gene sets based on overrepresentation of functionally coherent subnetworks within the graph (Figures S1, S2, S3, S4, S5, S6, S7, S8 and S9). One such subnetwork implicated prenylation of Rab-family GTPases in support of H1N1 replication (Figure S4 and Figure 3D). To test this we employed 3-IPEHPC, a specific inhibitor of the type II Geranylgeranyl-transferases (IC50 of 1.27 μM and a K_i_ of 0.211 μM for Rab1a modification [29]). As such, 3-IPEHPC specifically inhibits modification of Rab-family proteins with a carboxy-terminal CC motif as opposed to the carboxy-terminal CAAX motif [29]. HBEC-30 cells pretreated with 3-IPEHPC for 24 hours were significantly refractory to infection by H1N1 (Figure 5A, B). Inhibitory activity was observed at concentrations as low as 125 nM (Figure 5C). Unlike SB218078, A549 cells were also responsive to 3-IPEHPC (Figure 5D, E). While the use of a mouse-adapted virus facilitates large-scale screening and allows comparisons with other published screening efforts, the extent to which results translate to seasonal or highly pathogenic strains is not established. Importantly, 3-IPEHPC was protective against infection with the avian strain H5/N1 and the recent pandemic swine flu strain H1/N1 (Figure 5D, E).

**Figure 6:**
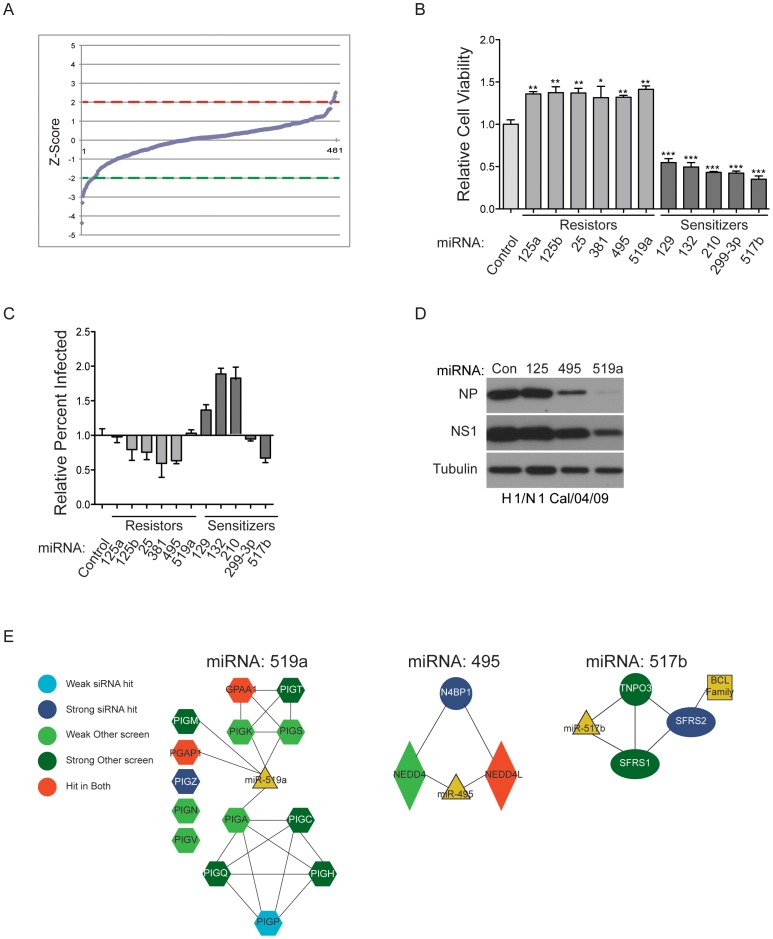
miRNA Screen. (A) HBEC30s were transfected with miRNA mimics and screened using conditions identical to the siRNA screen. Z-Scores were calculated for individual oligos and plotted according to rank order. Dashed lines indicate 2 standard deviations above (red) and below (green) the mean of the distribution. (B) HBEC30s were transfected with selected miRNA mimics, infected with WSN and cell viability phenotype was measured 48 hours post infection. (C) Cells treated as in B were fixed and immunostained for viral protein 12 hours post infection. (D) A549 cells were transfected with miRNA mimics and infected with pandemic H1/N1. Cell lysates were collected 24 hours post infection and viral proteins were detected by immunoblotting. (E) Network analysis of miRNA predicted targets. Node behavior in siRNA screens is indicated. Edges indicate physical or functional interactions among nodes. (P values; * <0.05, ** <0.01, *** <0.0001).

A stark limitation of arrayed siRNA screens is the requirement for “single gene” phenotypic penetrance. This can limit sensitivity of detection of relevant molecular entities due to insufficient protein depletion and/or the presence of functionally redundant gene products. As a mechanism to potentially reveal combinatorial contributions of gene function to viral replication and cytopathic effects, we repeated the original screen using a library of 426 human microRNA mimics. These reagents have the advantage of inducing multigenic perturbations, though accurate assignment of target space is a significant challenge. This effort identified a small cohort of miRNA mimics that either enhanced or deflected H1N1-induced cell death (Figure 6A, B, Table 6 in Supporting Information S1). 11 of these were further examined for consequences on H1N1 viral protein production in HBEC30 cells, which identified both sensitizers and resistors that enhanced or repressed viral replication (Figure 6C). Of note, a test for “hits” that also have activity against the recent pandemic strain Cal/04/09 identified two miRNA mimics that impair Cal/04/09 protein production in A549 cells (hsa-miR-495 and hsa-miR-519a, Figure 6D). To infer biological processes that may be engaged by the miRNAs that can impair H1N1 replication, we examined the intersection of predicted miRNA targets and single-gene perturbations that behaved similarly to the subject miRNA. Candidate miRNA target genes were selected based on seed sequence presence in 3′ UTRs as defined by Target Scan context scores. These predictions were intersected with siRNA data from this study and those of the 4 whole-genome siRNA screens that measured influenza virus replication [4]–[7]. When considered as a heuristic, this analysis produced three subnetworks that may correspond to the miRNA mode of action, namely the glycosylphosphatidylinositol transamidase, viral and host protein ubiquitylation [30], [31] and alternative mRNA splicing (Figure 6E).

Here we have focused on isolation of H1N1 pathogenicity response modifiers in human bronchial airway epithelial cells (HBEC). This cell type was selected as tissue culture model that may be enriched for conservation of cell autonomous biological features representative of the viral target tissue. These cells resist plaque formation, but are highly sensitive to single cycle infection. From whole-genome siRNA and miRNA mimic screening, both candidate sensitizer and resistor response modifiers were identified. A key deliverable from this analysis was the identification of gene products that apparently serve to restrain cell death responses that would otherwise engage upon detection of viral infection. Though not required to support cell viability in the absence of viral challenge, depletion of genes in this class enhanced the death response to H1N1 infection concomitant with restraining H1N1 protein production. As such, this class may represent targets for interventions that restrain propagation of multi-cycle infection by facilitating suicide of infected cells prior to production of new infectious particles. A chemically addressable member of this class, CHEK1, showed strong activity in multiple HBEC lines but not in A549, a non-small cell lung tumor derived line commonly employed to model influenza virus infection. This suggests that intervention targets may be available in normal epithelial cells that are uncoupled from host regulatory networks in cancer cells.

## Materials and Methods

### RGGT Inhibitor

Racemic 3-IPHPC (2-hydroxy-3-imidazo[1,2-a]pyridine-3-yl-2-phosphonopropionic acid) was prepared and characterized as described previously [32], [33] and stored at <0°C and pH ≥7 [32], [33]. The purity was ≥98%by ^1^H NMR. The inhibitor was tested in this work as the racemate [32], [33]. It was subsequently demonstrated that the individual enantiomers have markedly different IC_50_ and K*i* values for inhibition of Rab1a prenylation, thus the racemate value obtained here probably represents an upper limit with respect to the potency of the more active stereoisomer.

### Cell Culture

HBEC30-KT cells were cultured in KSFM (Invitrogen Cat#17005) with 1% Pen/strep antibiotics as previously described [34]. MDCK and A549 cells [from ATCC] were grown in DMEM with 10% FBS.

### Plaque Assay

5×10^5^ MDCK [from ATCC] and HBEC-30KT cells [34] were plated in 6 well plates and grown to confluence overnight. Cells were infected with WSN virus at 10 fold dilutions with a starting concentration of 10^8^ pfu/ml. Infected cells were allowed to incubate at 37°C with tilting every 10 minutes. After incubation liquid was aspirated and 2 ml of agar solution was added to wells and allowed to solidify for 1 min. Plates were incubated for 48 hrs at 37°C. Following incubation plates were fixed with formaldehyde for 1 hr. Fixative and agar was removed and cells were stained with crystal violet.

### Viral Protein Detection

HBEC30-KT cells [34] were plated in 96 well plates and incubated overnight. Cells were infected with WSN virus at an MOI 5. Whole cell lysates were collected at the indicated time point and separated by 12% SDS-PAGE gel and transferred to a nitrocellulose membrane. Cultures for immunofluorescence were fixed with 4% formaldehyde at indicated time point. Viral protein was detected in both cases with antibodies for pan influenza A (1∶200), M2 (1∶500) or NP (1∶500) proteins{α-tubulin (cellsignalling rabbit mAb, cat#2125S), anti-NS1 [35], anti-NP (Abcam, cat# ab20343)} followed by detection with either HRP conjugated secondary or staining with Alexa 498 (1∶5,000) or Alexa 594 (1∶5,000) conjugated secondary antibodies(from Invitrogen). Wells were imaged with a 20x lens on a BD Pathway 855 microscope. Imaged cells were segmented using Hoescht staining and distance from nucleus, αIVA fluorescence intensity was measured, with Attovision software.

### Viral Titers

HBEC30-KT cells were infected with WSN virus and supernatants were collected at 24 hours post infection. Supernatants were then added to MDCK cells at 1% final concentration and MDCKs were fixed 14 hours after supernatant addition and viral production in MDCK cells was detected immunostaining.

### H5N1 and H1N1 Pandemic Virus

A549 cells were infected with either either influenza A/Vietnam/1203/2004 (H5N1) HALo virus or pandemic influenza A/Cal/04/09 virus at an MOI of 1 and viral protein was detected by immunoblot.

### siRNA and miRNA Screens

The siRNA screen was performed using the Dharmacon library targeting 21,125 genes HBEC30 were plated into 96 well plates at 10,000 cells per well and siRNAs were reverse transfected. Each siRNA pool was transfected in two sets of triplicates for a total of 6 wells for each siRNA, three wells for infection with IVA and three wells for mock infection, with a concentration of 50 nM for oligos and 0.1% DharmaFECT 3 reagent. Cells were incubated for 48 hrs after transfection and infected with influenza A/WSN/33 (H1N1) virus at an MOI of 5. Forty-eight hours after infection cell viability was assayed using CellTiter-Glo, 15 μl of Promega's CellTiter-Glo was added to wells on a 96 well plate for a final concentration of 7.5%. Plates were rocked for two minutes followed by 10 minutes incubation. Luciferase activity was measured with a PerkinElmer EnVision reader. The miRNA mimic screen was performed with the Dharmacon miRNA mimic library corresponding to 426 human miRNA's. Screening conditions were identical to those described above with the exception of a 72-hour incubation between transfection and infection.

### Data Normalization and Z-Score Calculation

To remove position effects, raw values from each well were normalized to the median well of their respective row using the siMacro found at (http://sourceforge.net). To control for contamination and technical issues the top 5% of outliers with the highest coefficient of variation among triplicates were removed. Outliers were defined as wells with the largest distance among triplicate values. Normalized data was log2 transformed for proper distribution of sensitizers and resistors and a ratio of infected over mock infected was obtained. To control for batch effects, Z-Scores were calculated using batch specific variance where for each siRNA pool i Zi =  xi-µbatch/σbatch, where x is the raw data to be normalized, μ is the mean of the batch population, and σ is the standard deviation of the batch population.

### Functional Class Assignment

Published data sets were obtained from four siRNA screens for influenza A modulators that used viral replication as an end point assay [4]–[7]. Candidate hits in our screen were queried for behavior as regards viral replication. Hits that modulated viral replication greater than 1.5 standard deviations were assigned to functional classes. In cases were hits showed multiple phenotypes the strongest phenotype was used for classification.

### Data set Comparisons

Screening data was compared for overlap with published hit lists for cell cycle regulators [36], [37], host regulators of HIV infection [38]–[40], and interferon stimulated genes (ISGs) [41]. Published hits that correlated with a change in cell viability greater than two standard deviations were considered as positive hits.

### miRNA Predicted Networks

Predicted targets of miRNAs based on seed sequence were obtained from TargetScan (http://targetscan.org). Network analysis of predicted hits was completed using Ingenuity IPA (http://ingenuity.com) and queried for behavior in siRNA screens for regulators of influenza A infection.

### Network Analysis

Z-scores were used as weights for NetWalk analysis [28]. Interactions with 350 highest and 350 lowest Edge Flux values were used to construct the networks with high and low z-scores, respectively. Analyses and graphics were done in the NetWalker desktop application (Komurov et al, manuscript submitted, http://research.cchmc.org/netwalker).

### Compounds

HBEC30 or A549 cells were plated on 96 well plates overnight. Media was removed and replaced with media containing SB218078 (1µM, 100nM 10nM) 3-IPEHPC (12.5µM, 1.25µM, 125nM) DMSO (0.06%) or plain media. Cells were incubated overnight and then infected with WSN at an MOI of 5. Cells were fixed with 4% formaldehyde at 8 hours, 12 hours and 24 hours post infection and stained as described previously. SB218078 was purchased from Tocris biosciences cat # 2560 and dissolved in DMSO. 3-IPEHPC was dissolved in PBS.

## Supporting Information

Figure S1 **Individual siRNA oligo assays.** HBEC30 cells were transfected in triplicate with four individual siRNA oligos and infected with WSN. Cell viability was measured 48 hours post infection and a two-tailed Student's t-test was performed to determine significance. Green boxes are oligos with a p value less than 0.05.
(PDF)

Figure S2 **Network analysis of siRNA screen results.** Data from siRNA screen results was used for NetWalk analysis. Nodes are colored based on Z-Score with red for positive and green for negative, edges are colored based on interactions, PPI: protein-protein interaction, TF-Target: gene regulation, GO: GO similarity. Networks analysis was performed with entire data set.
(PDF)

Figure S3 **Networks analysis was performed with resistors all edges.**
(PDF)

Figure S4 **Networks analysis was performed with resistors for signaling edges.**
(PDF)

Figure S5 **Networks analysis was performed with resistors with gene regulation edges.**
(PDF)

Figure S6 **Networks analysis was performed with sensitizers all edges.**
(PDF)

Figure S7 **Networks analysis was performed with sensitizers signaling edges.**
(PDF)

Figure S8 **Networks analysis was performed with sensitizers gene regulation edges.**
(PDF)

Figure S9 **Networks analysis was performed with and sensitizers GO similarity edges.**
(PDF)

Supporting Information S1 **Supplementary tables.**
(XLS)
