# Supplementary figures and images for: Host Modulators of H1N1 Cytopathogenicity
Source: PLoS One. 2012 Aug 2;7(8):e39284. doi: 10.1371/journal.pone.0039284 (PMC3410888; doi:10.1371/journal.pone.0039284)

Figure S1

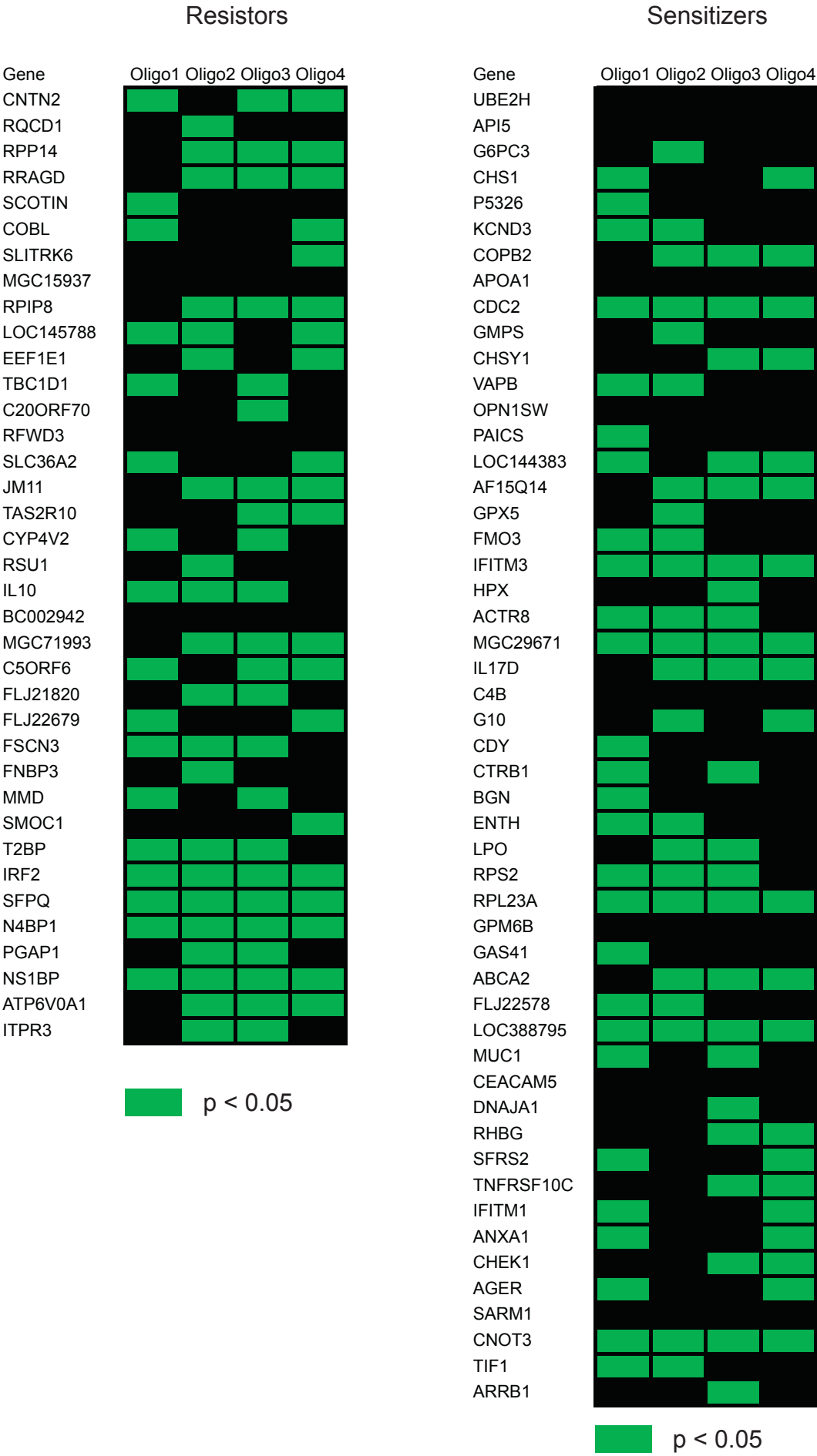

Supplement: Figure S1 — Individual siRNA oligo assays. HBEC30 cells were transfected in triplicate with four individual siRNA oligos and infected with WSN. Cell viability was measured 48 hours post infection and a two-tailed Student's t-test was performed to determine significance. Green boxes are oligos with a p value less than 0.05. (PDF) [file pone.0039284.s001.pdf]

Figure S2

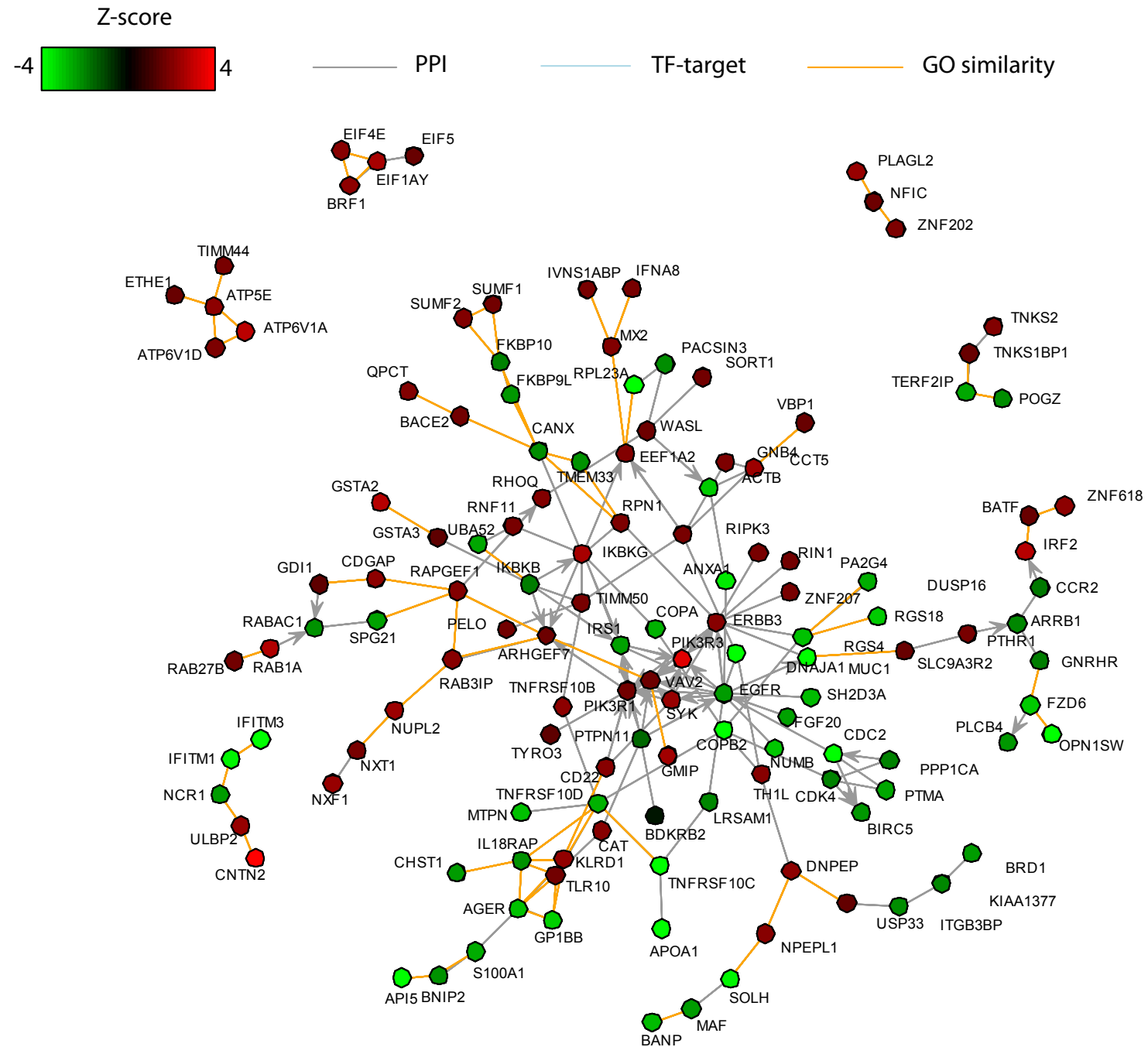

Supplement: Figure S2 — Network analysis of siRNA screen results. Data from siRNA screen results was used for NetWalk analysis. Nodes are colored based on Z-Score with red for positive and green for negative, edges are colored based on interactions, PPI: protein-protein interaction, TF-Target: gene regulation, GO: GO similarity. Networks analysis was performed with entire data set. (PDF) [file pone.0039284.s002.pdf]

Z-score

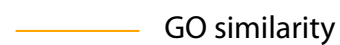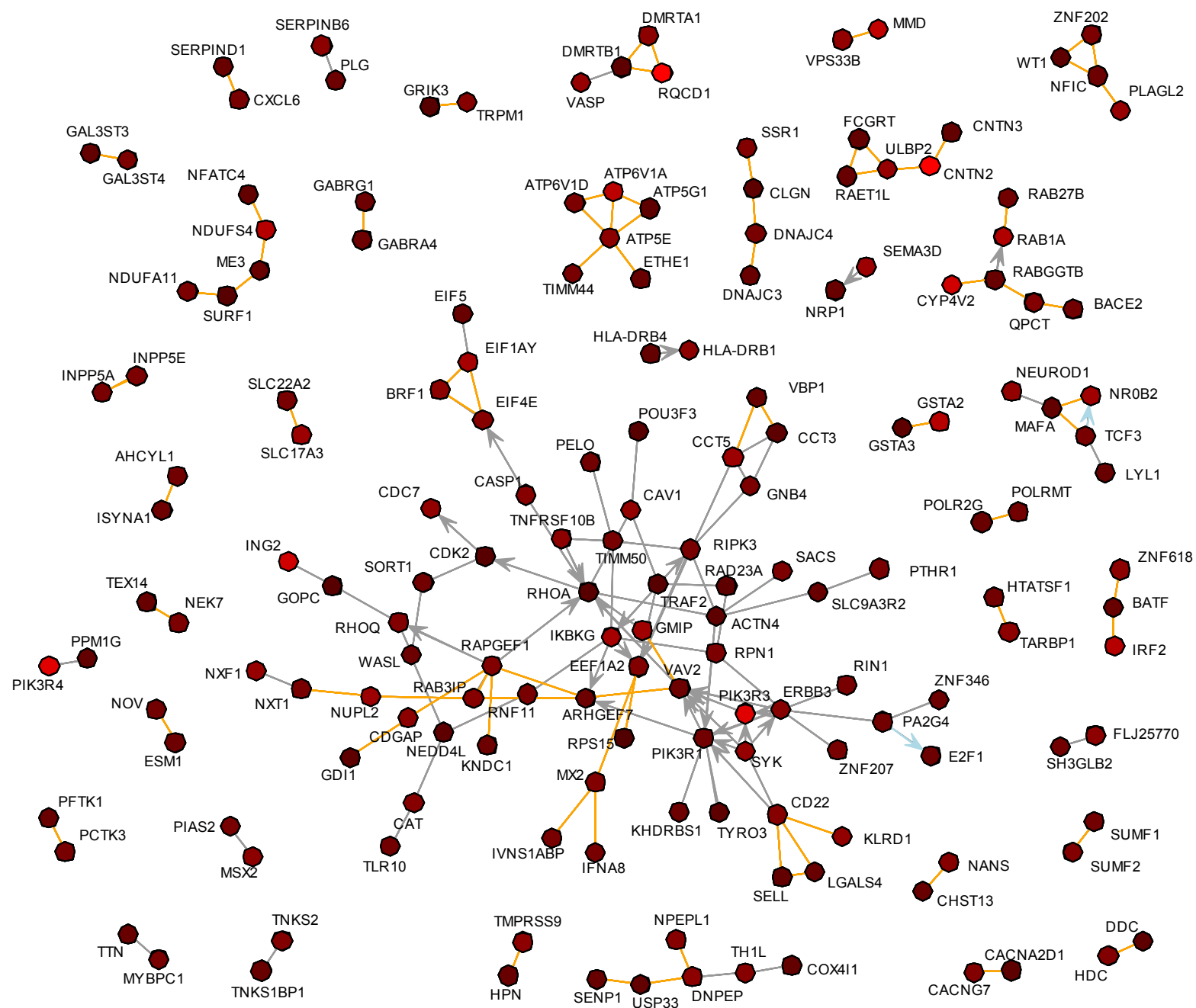

Supplement: Figure S3 — Networks analysis was performed with resistors all edges. (PDF) [file pone.0039284.s003.pdf]

Figure S4

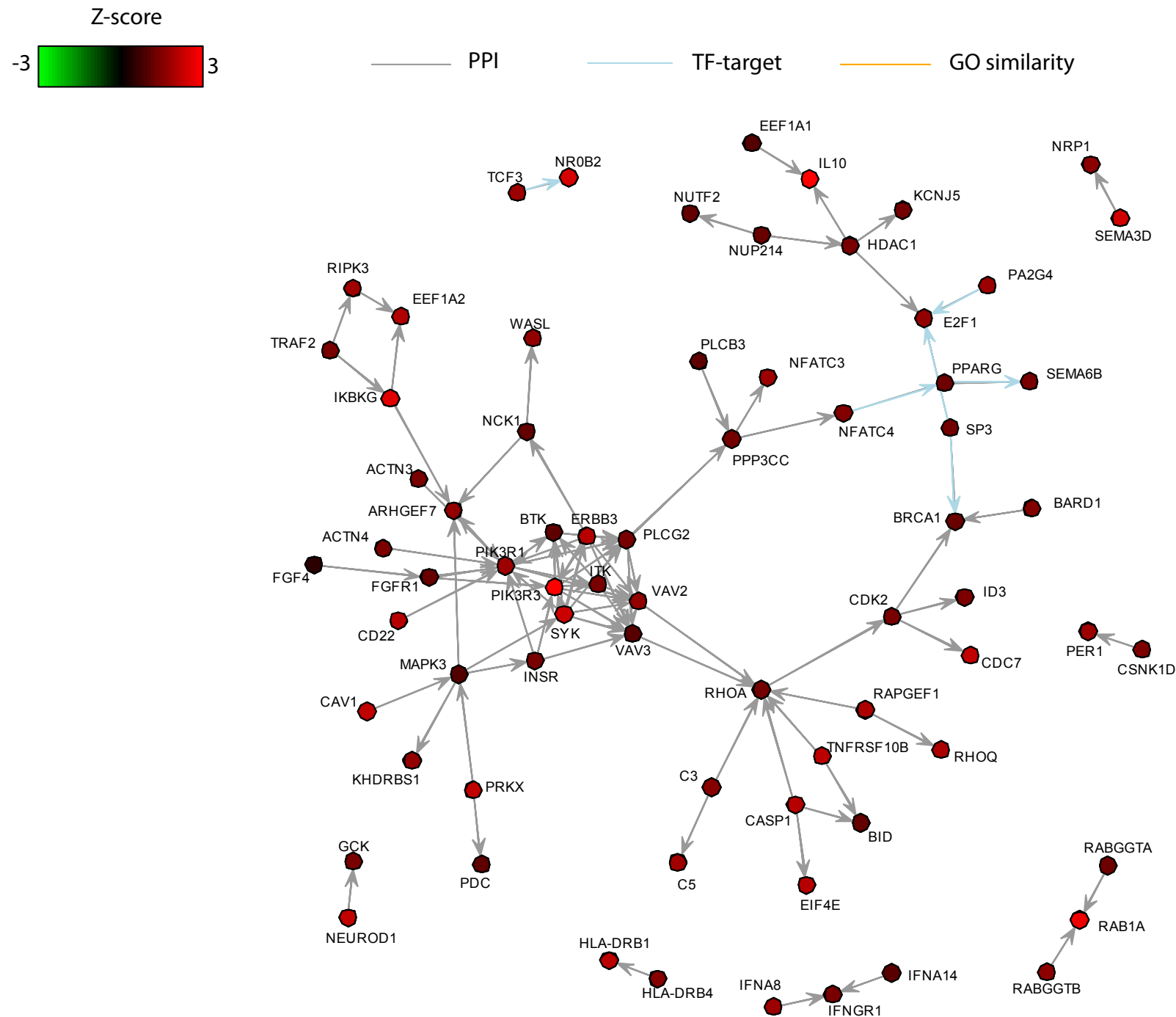

Supplement: Figure S4 — Networks analysis was performed with resistors for signaling edges. (PDF) [file pone.0039284.s004.pdf]

Figure S5

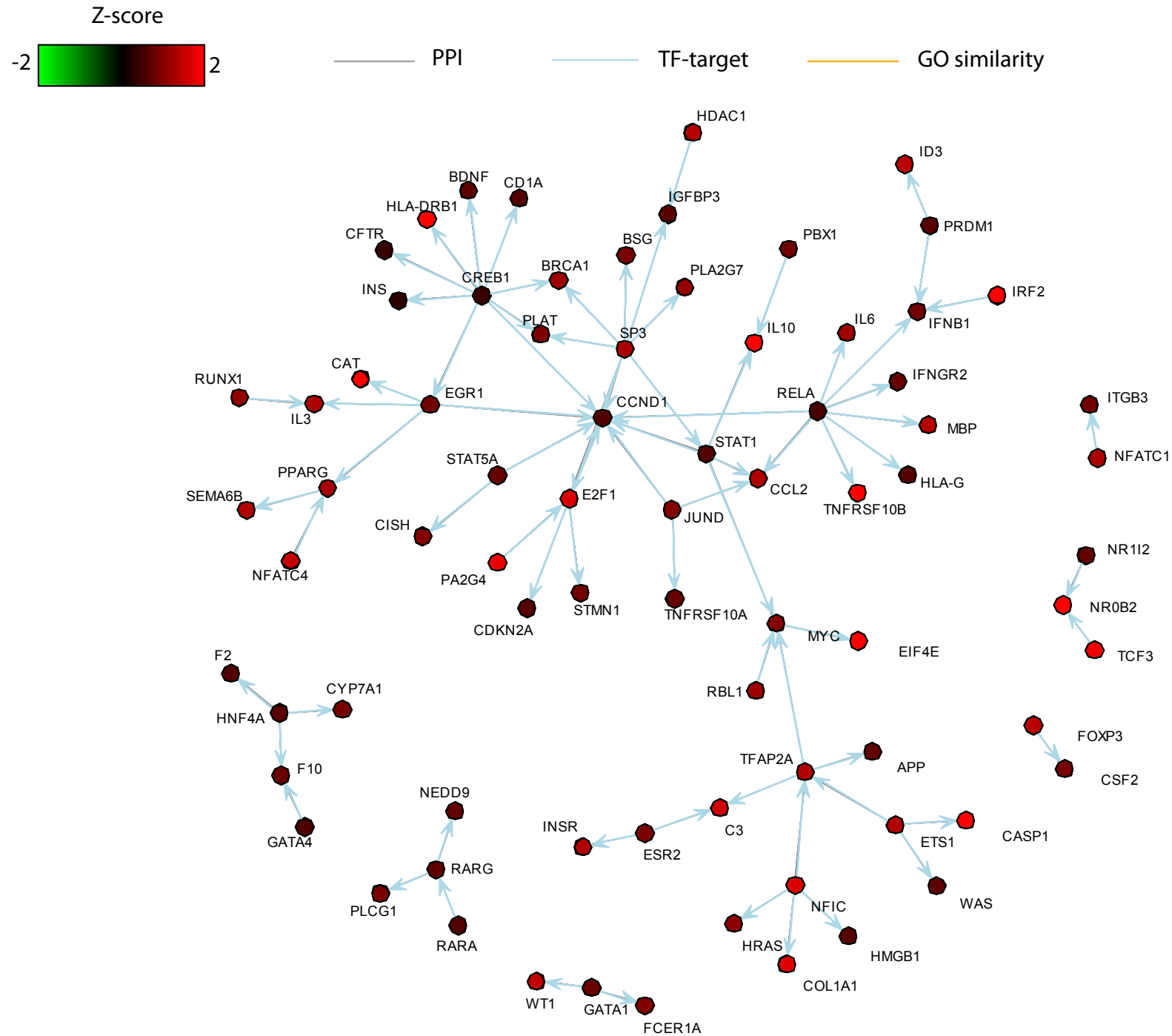

Supplement: Figure S5 — Networks analysis was performed with resistors with gene regulation edges. (PDF) [file pone.0039284.s005.pdf]

Figure S6

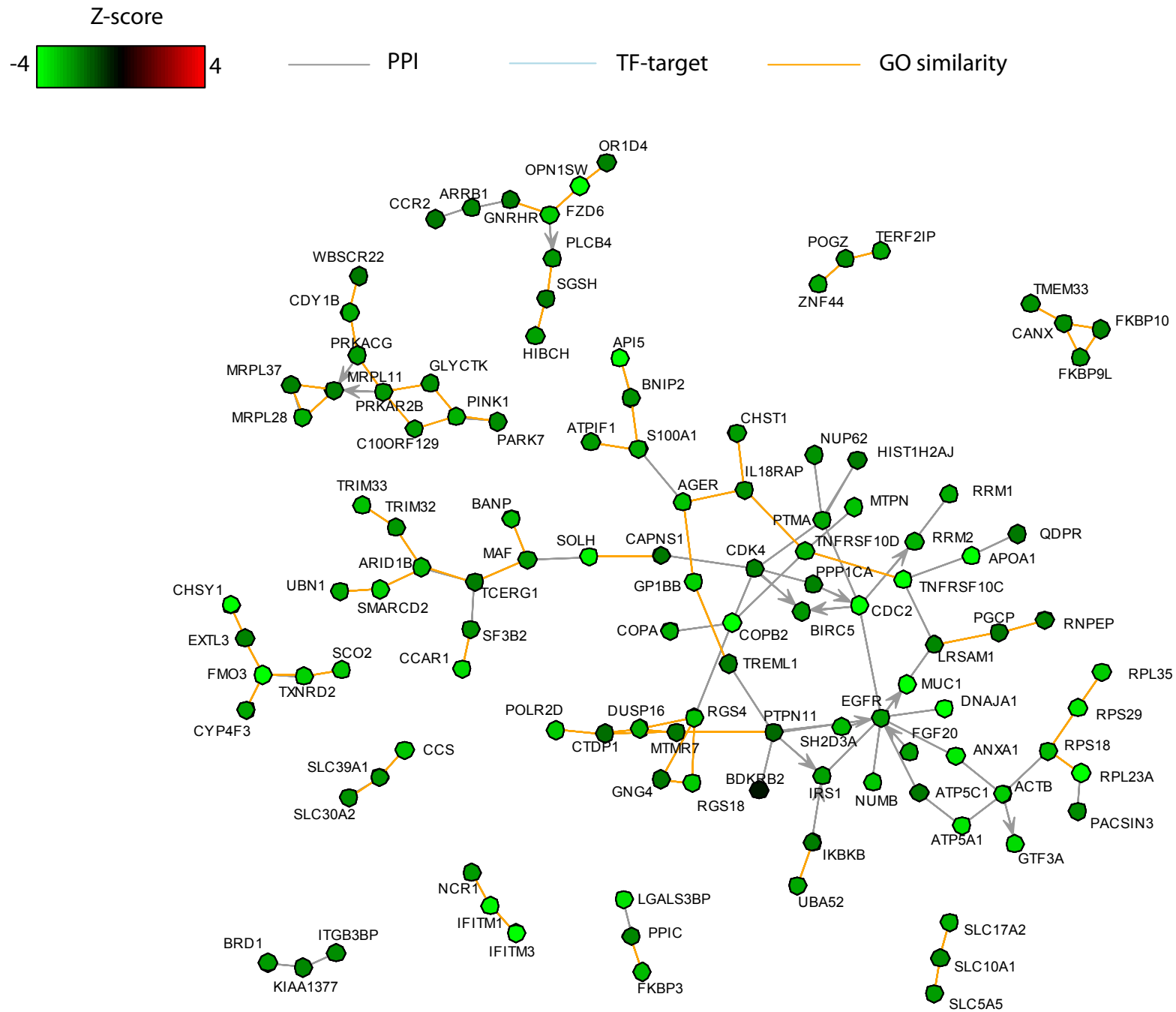

Supplement: Figure S6 — Networks analysis was performed with sensitizers all edges. (PDF) [file pone.0039284.s006.pdf]

Figure S8

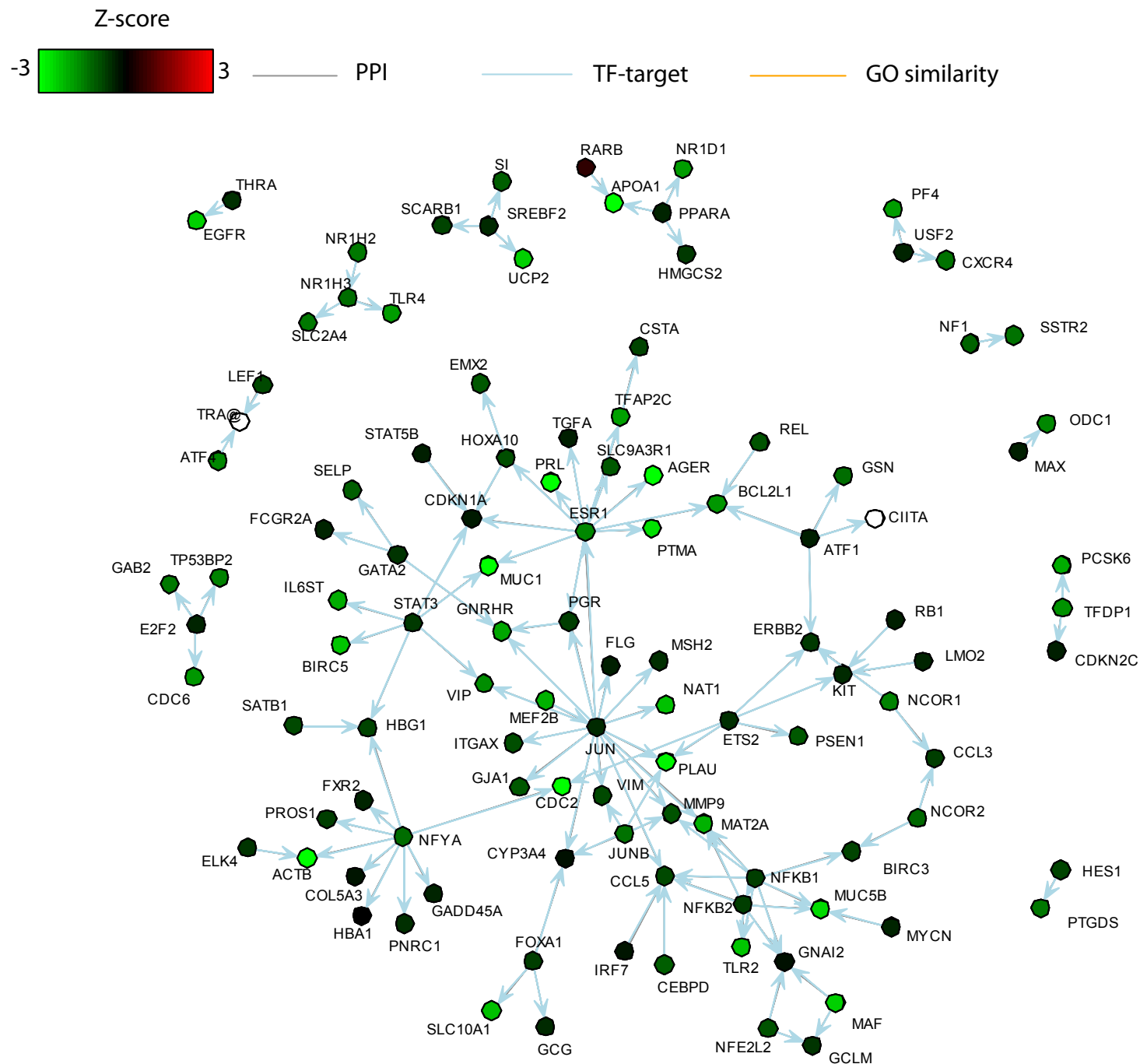

Supplement: Figure S8 — Networks analysis was performed with sensitizers gene regulation edges. (PDF) [file pone.0039284.s008.pdf]

Figure S9

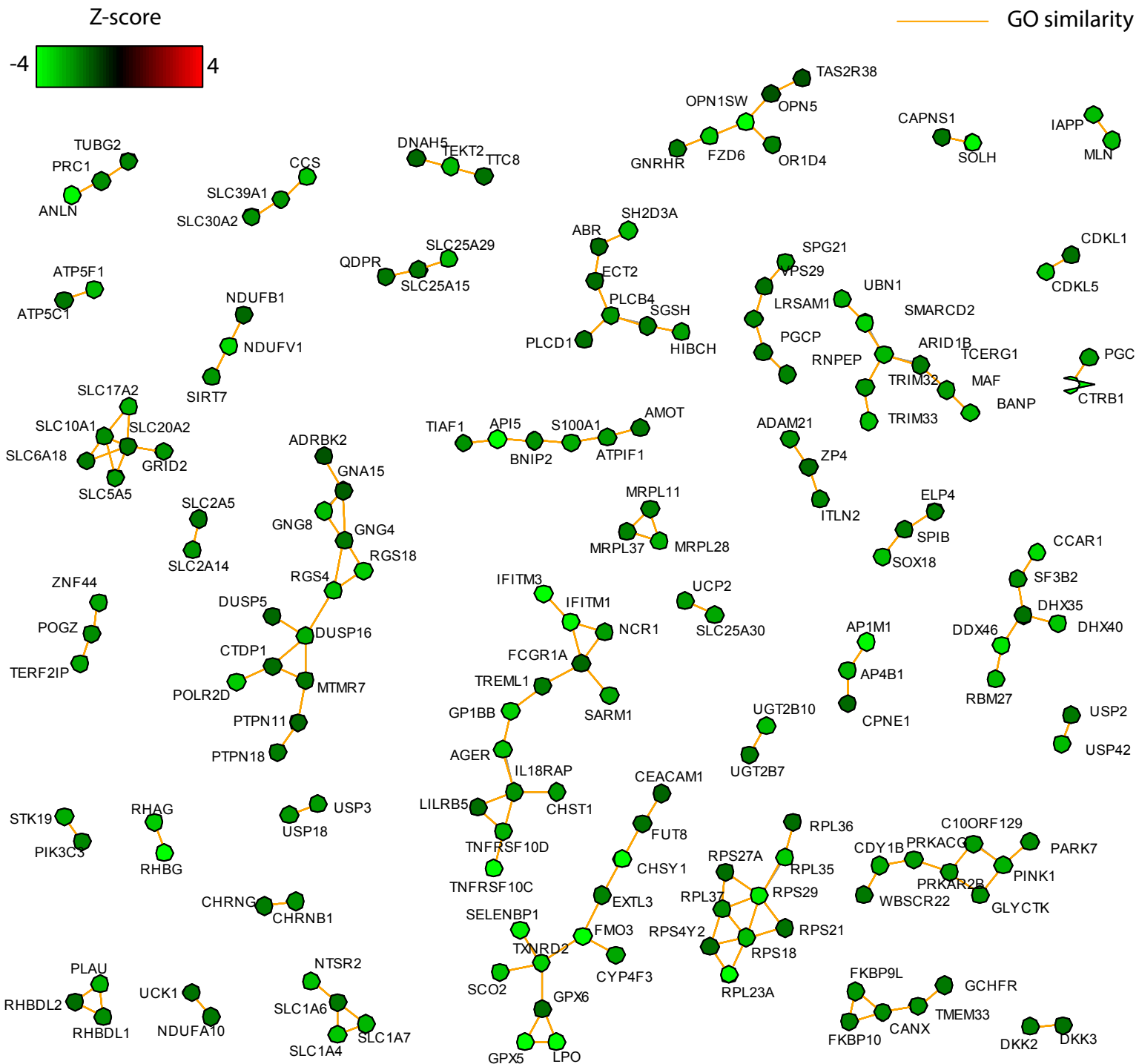

Supplement: Figure S9 — Networks analysis was performed with and sensitizers GO similarity edges. (PDF) [file pone.0039284.s009.pdf]
