# Supplementary material for: Host Modulators of H1N1 Cytopathogenicity
Source: PLoS One. 2012 Aug 2;7(8):e39284. doi: 10.1371/journal.pone.0039284 (PMC3410888; doi:10.1371/journal.pone.0039284)
Supplement: Figure S7 — Networks analysis was performed with sensitizers signaling edges. (PDF) [file pone.0039284.s007.pdf]

Figure S7

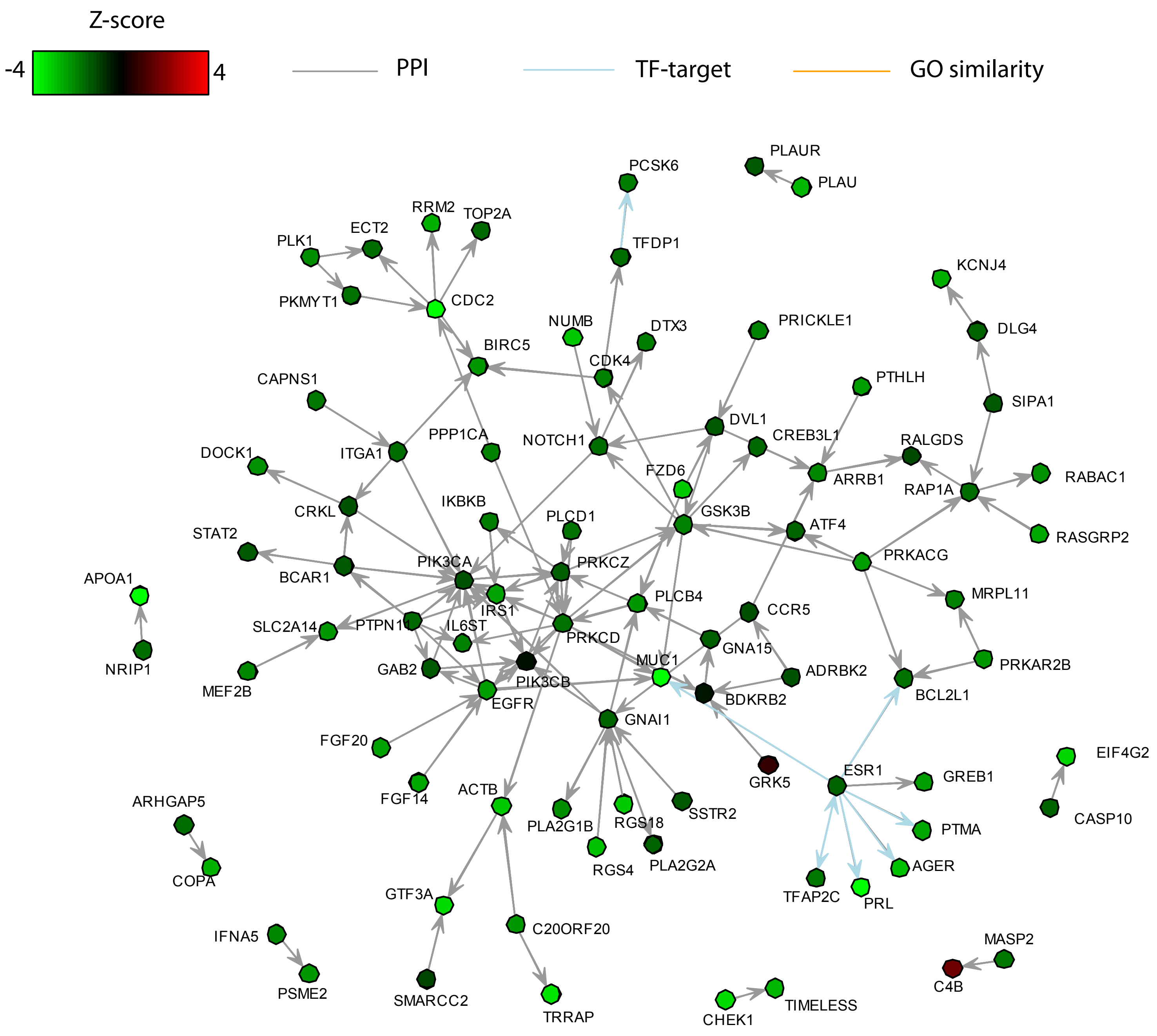

Z-score
-4
4
PPI
TF-target
GO similarity
PLAUR
PLAU
PCSK6
TFDP1
RRM2
TOP2A
ECT2
PLK1
PKMYT1
CDC2
KCNJ4
DLG4
NUMB
DTX3
PRICKLE1
BIRC5
CDK4
CAPNS1
PTHLH
SIPA1
DVL1
NOTCH1
PPP1CA
CREB3L1
ITGA1
DOCK1
RALGDS
FZD6
ARRB1
RABAC1
RAP1A
CRKL
IKBKB
GSK3B
PLCD1
ATF4
RASGRP2
STAT2
PIK3CA
PRKCZ
PRKACG
BCAR1
APOA1
MRPL11
IRS1
PLCB4
CCR5
SLC2A14
PTPN11
IL6ST
PRKCD
GNA15
NRIP1
ADRBK2
PRKAR2B
MUC1
GAB2
PIK3CB
MEF2B
BDKRB2
BCL2L1
EGFR
GNAI1
FGF20
EIF4G2
ESR1
GRK5
GREB1
FGF14
ACTB
SSTR2
ARHGAP5
CASP10
PLA2G1B
RGS18
PTMA
RGS4
PLA2G2A
AGER
COPA
GTF3A
TFAP2C
PRL
C20ORF20
IFNA5
MASP2
C4B
PSME2
SMARCC2
TRRAP
TIMELESS
CHEK1
